# Cardiac Characteristics of Hospitalized Influenza Patients: An Interim Analysis From the FluHeart Study

**DOI:** 10.1111/irv.70067

**Published:** 2025-02-18

**Authors:** Kristoffer Grundtvig Skaarup, Filip Soeskov Davidovski, Emil Durukan, Daniel Modin, Mats Christian Højbjerg Lassen, Maria Dons, Anne Marie Reimer Jensen, Niklas Dyrby Johansen, Morten Sengeløv, Frederikke Vyff, Nino Emanuel Landler, Gorm Boje Jensen, Anne Bjerg Nielsen, Jacob Christensen, Raphael Hauser, Peter Schnohr, Rasmus Møgelvang, Lene Nielsen, Jens‐Ulrik Stæhr Jensen, Tor Biering‐Sørensen

**Affiliations:** ^1^ Department of Cardiology Copenhagen University Hospital ‐ Herlev and Gentofte Copenhagen Denmark; ^2^ Center for Translational Cardiology and Pragmatic Randomized Trials, Department of Biomedical Sciences, Faculty of Health and Medical Sciences University of Copenhagen Copenhagen Denmark; ^3^ The Copenhagen City Heart Study Copenhagen University Hospital ‐ Bispebjerg and Frederiksberg Copenhagen Denmark; ^4^ Department of Cardiology Copenhagen University Hospital Copenhagen Denmark; ^5^ Department of Clinical Microbiology Copenhagen University Hospital ‐ Herlev and Gentofte Copenhagen Denmark; ^6^ Respiratory Medicine Section, Department of Medicine Copenhagen University Hospital – Herlev and Gentofte Copenhagen Denmark; ^7^ Steno Diabetes Center Copenhagen Copenhagen Denmark

**Keywords:** cardiac function, COVID‐19, influenza infection

## Abstract

**Background:**

Influenza infection has been associated with multiple cardiac complications including acute heart failure and myocardial infarction. The FluHeart study aims to uncover the potential effect of influenza infection on cardiac structure and function as assessed by echocardiography during hospitalization.

**Methods:**

This prospective cohort study included hospitalized influenza patients of the 2021–2022 influenza season. Participants underwent echocardiography using a prespecified protocol. Participants were successfully matched 1:1:1 on age, sex, and heart failure status with controls from the general population and controls hospitalized with COVID‐19.

**Results:**

This interim analysis involved 108 participants (36 influenza patients, 36 general population controls, and 36 COVID‐19 patients). Mean age was 72 ± 18 years and 58% were male. Median time from admission to echocardiography was 1 day (IQI: 1:1) for influenza patients. The prevalence of left ventricular (LV) dysfunction was 75%, and right ventricular (RV) dysfunction was observed in 20% of influenza patients. N‐terminal pro‐brain natriuretic peptide levels were elevated ≥ 300 pg/mL in 62%, and 19% exhibited myocardial injury with elevated high‐sensitivity troponin I levels. RV tricuspid annular plane systolic excursion and LV early diastolic peak mitral inflow to early diastolic tissue velocity were significantly worse in influenza patients compared to general population controls. Echocardiographic measures did not significantly differ between patients hospitalized with influenza and COVID‐19.

**Conclusion:**

In this interim analysis of the FluHeart study, both RV and LV function measures were significantly impaired in hospitalized influenza patients compared with matched general population controls. The extent of impairment resembled that observed in hospitalized COVID‐19 patients.

## 1 Introduction

For nearly a century, influenza infection has been linked to an elevated incidence of cardiovascular mortality [1]. During the last two decades, there has been an increasing emphasis on understanding the risk of cardiovascular complications during acute influenza. Among hospitalized influenza patients, acute heart failure has been reported as the most common cardiovascular event [2]. Furthermore, influenza has been associated with an increased risk of myocardial infarction, arrhythmia, stress cardiomyopathy, myocarditis, and pericarditis [3]. Influenza infection may impact both right (RV) and left (LV) ventricular function through a combination of systemic conditions induced by the inflammatory response and specific effects of influenza virus [4]. Influenza‐associated cardiovascular morbidity and mortality have mostly been established through large epidemiological studies [5, 6, 7, 8]. In addition, several clinical studies have reported myocardial injury assessed by biochemical biomarkers to be prevalent during influenza infection [9, 10, 11, 12]. Echocardiography enables direct assessment of the cardiac mechanisms during acute influenza infection. However, investigations of the prevalence of myocardial dysfunction during acute influenza infection have been limited in previous studies [12, 13]. Hence, further prospective investigations are necessary to provide a comprehensive understanding of cardiac mechanics during acute influenza infection.

The objective of the FluHeart study is to examine the effects of influenza infection on cardiac function in hospitalized influenza patients. In this interim analysis of the first influenza season of the study, we sought to determine the prevalence of cardiac dysfunction using echocardiography and biomarkers. Additionally, we aimed to compare these findings with matched controls from both the general population and a sample of hospitalized COVID‐19 patients.

## 2 Methods

### 2.1 Population

The FluHeart study is a prospective cohort study of hospitalized patients with laboratory‐confirmed influenza infection at Copenhagen University Hospitals Herlev and Gentofte. The FluHeart study commenced enrollment during the influenza season 2021/2022 and concluded by May 2024. The present interim analysis included participants enrolled during the 2021/2022 influenza season. Inclusion criteria were (1) hospitalization with a laboratory‐confirmed diagnosis and (2) age ≥ 18 years. Exclusion criteria were (1) simultaneous infection with laboratory‐confirmed COVID‐19; (2) persons unable to understand, sign, or cooperate to informed consent; and (3) pregnancy. Departments were assessed daily, and patients with laboratory‐confirmed influenza were approached. The investigators did not have any prior knowledge of the health status of the potential participants. Subsequently, the patients were screened for inclusion and exclusion criteria and were invited to participate independently of their prior health status. The study was approved by the regional ethics board and was conducted in accordance with the 2nd Declaration of Helsinki. The FluHeart Study is registered at Clinicaltrials.gov (NCT05084846).

### 2.2 Data Collection

All participants responded to a questionnaire concerning demographic information, vaccination status, previous infections, and cardiovascular risk factors such as family history of cardiovascular disease and smoking status. Clinical data including vital signs from the day of echocardiography, medicine use, and medical history were retrieved from electronic medical records. Diabetes was defined as the use of antidiabetic medication or reported in the electronic health record. Previous ischemic heart disease was defined as history of myocardial infarction, percutaneous coronary intervention, or coronary bypass grafting. Blood was retrieved on the day of inclusion for biochemical assessment of high‐sensitivity troponin I (hs‐TnI), N‐terminal pro‐brain natriuretic peptide (NT‐proBNP), and c‐reactive protein (CRP).

### 2.3 Echocardiography

Bedside transthoracic echocardiographic examinations were performed according to an extensive predefined protocol using portable Vivid iq Ultrasound Systems (GE Healthcare, Horten, Norway). The commercially available postprocessing software EchoPAC Version 206 (GE Vingmed Ultrasound AS, Horten Norway) was used to analyze all echocardiographic examinations. All echocardiograms were analyzed by a single trained investigator blinded to clinical and follow‐up information.

Echocardiographic parameters were measured, and abnormal values were defined according to current guidelines [14]. LV chamber dimensions including the interventricular septal thickness, LV internal diameter, and LV posterior wall thickness were measured in the parasternal longitudinal axis view in end‐diastole. LV size index was obtained by indexing the LV internal diameter to body surface area, and LV mass index was calculated with Devereux's formula [15]. Using pulsed‐wave Doppler in the apical four‐chamber view, early peak inflow velocity was sampled at the tips of the mitral valve leaflets, which was indexed to the mean of the early peak tissue velocities measured in the septal and lateral mitral annular segments (E/e′) of the LV with pulsed‐wave tissue Doppler imaging. LV ejection fraction (LVEF) was measured with Simpson's biplane method in the apical four‐ and two‐chamber views. Left atrium (LA) volumes were measured in the apical four‐chamber and two‐chamber views using the biplane area‐length method, and LA volume index was calculated. Tricuspid annular plane systolic excursion (TAPSE) was measured with M‐mode in an apical four‐chamber view focused on the RV. Tricuspid regurgitation peak velocity (TR V_max_) was assessed with continuous‐wave Doppler imaging with a sample placed in the regurgitant jet if present in the same view. Global longitudinal strain (GLS) of the LV was acquired by 2D‐speckle tracking echocardiography performed in apical four‐chamber, two‐chamber, and three‐chamber views optimized for speckle tracking. A semiautomated function traced the endocardial border, which subsequently defined a region of interest covering the LV wall. The location and width of the regions of interest could be adjusted or excluded by the investigator if deemed necessary. The LV myocardial wall was divided into six regional segments in each view, and GLS was calculated as the mean of the peak systolic value of all segments. RV free wall longitudinal strain (RVLS) was obtained by 2D‐speckle tracking analysis of a modified apical four‐chamber view focused on the three RV free wall segments and the mean of the peak values of each segment was calculated. All strain values are reported as absolute values. Any LV dysfunction was defined as at least abnormal LVEF (< 52%/54% for males and females respectively), GLS (< 16%), or E/e′ (> 14). Any RV dysfunction was defined as at least abnormal RLVS (< 20%), TAPSE (< 1.7 cm), or TR V_Max_ (> 2.8 m/s).

### 2.4 Controls and Matching

Two control groups were included in this study. The first control group consisted of individuals from the general population of the 5th Copenhagen City Heart Study [16]. The second control group consisted of hospitalized patients with laboratory‐confirmed COVID‐19 infection from the ECHOVID‐19 study [17]. For both comparisons, influenza patients were matched 1:1 with controls on sex, age (10‐year age intervals), and heart failure status. Both studies were performed by our laboratory and have been described in detail elsewhere [16, 17]. In brief, the 5th Copenhagen City Heart Study (*n* = 4465) is a prospective cohort study of the general population included from 2011 to 2015. All participants were examined with echocardiography, answered a self‐administered questionnaire, and had blood work performed (not troponins or NT‐proBNP). The echocardiographic examinations were performed with Vivid 9 ultrasound systems and analyzed offline with EchoPAC Version 113.1.5. The ECHOVID‐19 study (*n* = 305) is a prospective cohort study of admitted COVID‐19 patients at hospitals in Eastern Denmark from 2020 to 2021. These patients were examined with echocardiography, questionnaires, and bloodwork (including NT‐proBNP and troponins). Echocardiography was performed with a portable Vivid iq system and analyzed with EchoPAC Version 203. For both control groups, all echocardiographic parameters examined were analyzed by the same method in each study; thus, if a parameter were obtained differently originally, a trained investigator remeasured the parameter in question with the methods used in the FluHeart study. Troponin types and assays varied among hospitals in the ECHOVID‐19 study. To facilitate comparisons with the FluHeart study, troponin levels were categorized based on the upper limit of normality specific to each assay. NT‐proBNP levels were categorized using a clinical cutoff value of ≥ 300 pg/mL, in addition to the age‐based rule‐in values recommended by the ESC guidelines for acute heart failure [18].

### 2.5 Statistics

STATA SE 17.0 was used for all statistical analysis. Statistical significance was defined as a two‐tailed *p* < 0.05. Gaussian variables were expressed as means ± standard deviations and compared using two‐sample *t*‐tests. Non‐Gaussian distributed variables were presented as medians with interquartile intervals (IQI) and compared using the Kruskal–Wallis test. Categorical variables were listed as frequencies with percentages and compared using Fisher's exact test. Pairs of cases and controls were excluded from the comparisons of echocardiographic cardiac function parameters if either observation had a missing value for the assessed parameter. Mean difference of echocardiographic cardiac function parameters, along with 95% confidence intervals, was calculated between FluHeart cases and each control group and visualized in forest plots.

## 3 Results

Test positivity rates for the Danish 2021/2022 influenza season increased significantly during Week 6 of 2022. The first week registering a positive rate ≥ 10% occurred in Week 10, which continued until Week 15. A total of 36 hospitalized influenza patients were included out of 49 approached. Patients were included between 21st of February 2022 and 22nd of April 2022. Mean age was 72 ± 18 years, and 58% were male. Age (77 ± 25 years, *p* = 0.43) and sex distribution (54% males, *p* = 1.00) did not differ significantly between participants and screen failures. All participants were infected with influenza virus A, and 21 (58.3%) were vaccinated prior to hospitalization. Time from admission to inclusion and examination was median 1 day (IQI: 1:1). Baseline characteristics of cases and the two control groups are listed in Table 1. In cases, the prevalence of any LV dysfunction was 75%, whereas any RV dysfunction was observed in 20% of the patients. Additionally, 19.2% of the cases had elevated hs‐TnI and NT‐proBNP levels were at least 300 pg/mL in 61.5%. Detailed information regarding the prevalence of abnormal cardiac findings and exact values observed among hospitalized influenza patients can be found in Table 2.

**TABLE 1:** Baseline characteristics of cases and controls.

	Influenza patients	General population	*p*	COVID‐19 patients	*p*
Number	36	36		36	
Male (%)	21 (58.3)	21 (58.3)	1.00	21 (58.3)	1.00
Age, years (SD)	71.7 ± 18.6	71.1 ± 17.5	0.88	71.9 ± 19.2	0.98
Body mass index, kg/m ^2^ (SD)	25.7 ± 5.6	27.0 ± 6.3	0.35	24.9 ± 5.7	0.58
Smoking status (%)					
Current	11 (30.6)	8 (23.5)	0.36	1 (3.3)	0.014
Former	15 (41.7)	11 (32.4)		15 (50.0)	
Never	10 (27.8)	15 (44.1)		24 (36.4)	
Antihypertensive medication (%)	21 (58.3)	17 (47.2)	0.35	21 (58.3)	1.00
Cholesterol‐lowering medication (%)	10 (27.8)	7 (19.4)	0.41	27 (75.0)	< 0.001
Diabetes (%)	6 (16.7)	4 (11.1)	0.50	9 (25.0)	0.38
Heart failure (%)	1 (2.8)	1 (2.8)	1.00	1 (2.8)	1.00
Previous ischemic heart disease (%)	3 (8.3)	6 (16.7)	0.29	2 (5.6)	0.64
Atrial fibrillation (%)	8 (22.2)	6 (16.7)	0.55	10 (27.8)	0.59
COPD/asthma (%)	12 (33.3)	1 (2.8)	0.001	8 (22.2)	0.29
Systolic blood pressure, mmHg (SD)	137.9 ± 21.9	152.0 ± 22.0	0.008	124.5 ± 18.5	0.007
Diastolic blood pressure, mmHg (SD)	74.2 ± 14.0	80.3 ± 12.0	0.052	73.0 ± 13.2	0.72
Heart rate during exam, beats/min (SD)	80.3 ± 15.3	70.6 ± 13.6	0.006	79.9 ± 15.0	0.81
Atrial fibrillation during exam (%)	2 (5.6)	2 (5.6)	1.00	0 (0)	0.15
Time from admission to exam, days (IQI)	1 (1; 1)			4 (2; 9)	< 0.001
Early warning score (IQI)	2 (0; 4)	—	—	2 (0; 3)	0.48
C‐reactive protein, mg/L (IQI)	32.0 (12.0; 99.0)	—	—	53.0 (29.0; 89.0)	0.33
eGFR, mL/min/1.73 m2 (IQI)	78.6 (50.6; 94.8)	74.3 (58.7; 92.1)	0.94	71.0 (43.8; 92.7)	0.48

Abbreviations: COPD, chronic obstructive pulmonary disease; eGFR, estimated glomerular filtration rate.

**TABLE 2:** Cardiac findings among hospitalized influenza patients.

	Prevalence of abnormal findings	Continuous value
Left ventricular function		
LVEF < 52/54% (males/females)	22/34 (64.7%)	54.5 (49.0; 59.0)
GLS < 16%	12/35 (34.3%)	17.4 (14.9; 19.6)
E/e′ > 14	8/35 (22.9%)	10.1 (8.4–13.1)
Any LV dysfunction ^a^	27/36 (75.0%)	—
Right ventricular function		
RVLS < 20%	2/19 (10.5%)	23.0 (21.0; 27.7)
TAPSE < 1.7 cm	2/34 (5.9%)	2.1 (1.8; 2.3)
TR V_max_ > 2.8 m/s	5/25 (20%)	2.1 (1.6; 2.7)
Any RV dysfunction ^b^	7/35 (20.0%)	—
Structural parameters		
LV mass index ≥ 115/95 g/m ^2^ (males/females)	6/29 (20.7%)	85.1 (63.7; 97.4)
LV size index ≥ 3.1/3.2 cm/m ^2^ (males/females)	0/29 (0%)	2.5 (2.3; 2.8)
LA volume index ≥ 32 mL/m ^2^	9/36 (25%)	23.6 (17.9; 31.3)
Cardiac biomarkers		
hs‐TnI, ng/L		
< ULN	21/26 (80.8%)	15 (11; 37)
ULN to 2*ULN	0/26 (0.0%)	—
> 2*ULN	5/26 (19.2%)	—
NT‐proBNP, pg/mL		
> 300 pg/mL	16/26 (61.5%)	618.2 (181; 1678.0)
< aHF RiV	19/26 (73.1%)	—
≥ aHF RiV to < 2*aHF RiV	5/26 (19.2%)	—
≥ 2*aHF RiV	2/26 (7.7%)	—

Abbreviations: aHF RiV, acute heart failure rule‐in value; E/e′, early diastolic peak mitral inflow velocity to early diastolic peak tissue velocity; GLS, global longitudinal strain; hs‐TnI, high‐sensitivity troponin I; LA, left atrium; LV, left ventricular; LVEF, Left ventricular ejection fraction; NT‐proBNP, N‐terminal pro‐brain natriuretic peptide; RV, right ventricular; RVLS, right ventricular free wall longitudinal strain; TAPSE, tricuspid annular plane systolic excursion; TR V_max,_ tricuspid regurgitation peak velocity; ULN, upper limit of normality.

^a^ Any LV dysfunction is defined as the composite of either abnormal LVEF, GLS, or E/e′.

^b^ Any RV dysfunction is defined as the composite of either abnormal RVLS, TAPSE, and TR V_Max_.

### 3.1 Comparison to Matched General Population Controls

**FIGURE 1:**
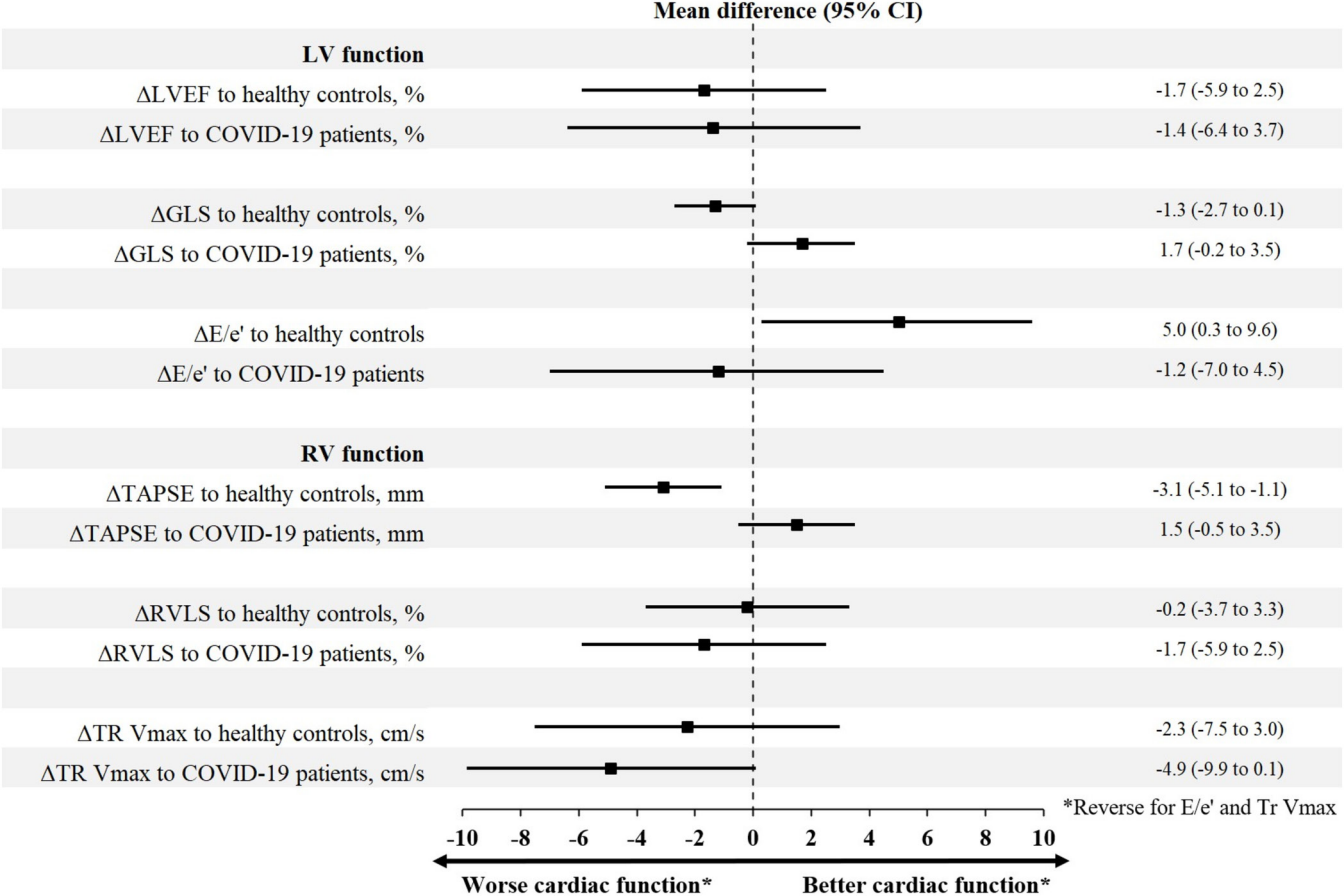
Mean difference in echocardiographic measures of cardiac function. Forest plots displaying mean differences in cardiac function assessed by echocardiography between influenza patients and matched general population controls and matched COVID‐19 patients. 95% confidence intervals are included. Mean differences were calculated by subtracting the values of controls from the values of influenza patients. E/e′, early diastolic peak mitral inflow velocity to early diastolic peak tissue velocity; GLS, global longitudinal strain; LV, left ventricular; LVEF, left ventricular ejection fraction; RV, right ventricular; RVLS, right ventricular free wall longitudinal strain; TAPSE, tricuspid annular plane systolic excursion; TR V_max,_ tricuspid regurgitation peak velocity.

Thirty‐six influenza patients were successfully matched 1:1 with 36 controls from the general population. Several notable differences were observed between these groups. Hospitalized influenza patients had a higher prevalence of chronic obstructive pulmonary disease or asthma, lower systolic blood pressure, and higher heart rate during echocardiography. LV diastolic function was significantly impaired in hospitalized influenza patients compared with controls as assessed by E/e′ (10.2 [IQI: 8.4; 14.1] vs. 8.8 [IQI: 6.6; 11.1], *p* = 0.026), but no difference was observed regarding the systolic measurements LVEF and GLS. Of the investigated RV function parameters, TAPSE was significantly lower in influenza cases vs. matched general population controls (2.1 cm [IQI: 1.8; 2.3] vs. 2.4 cm [2.2; 2.7], *p* = 0.001). Cardiac characteristics of influenza vs. healthy matched controls are listed in Table 3. Figure 1 illustrates the mean differences in echocardiographic parameters between cases and controls (Figure 1).

**TABLE 3:** Cardiac characteristics of influenza patients vs. healthy matched controls and matched hospitalized COVID‐19 patients.

	Influenza patients	General population	*p*
LV function			
LVEF, % ( *n* = 60)	54.0 (47.8; 59.2)	55.9 (48.8; 59.5)	0.65
GLS, % ( *n* = 70)	17.4 (14.9; 19.6)	18.7 (17.1; 20.0)	0.083
E/e′ ( *n* = 58)	10.2 (8.4; 14.1)	8.8 (6.6; 11.1)	0.026
RV function			
TAPSE, cm ( *n* = 68)	2.1 (1.8; 2.3)	2.4 (2.2; 2.7)	0.001
RVLS, % ( *n* = 38)	23.0 (21.0 27.7)	25.4 (19.8; 27.0)	0.61
TR V_max_ , m/s ( *n* = 26)	2.3 (1.7; 2.9)	2.4 (2.3; 2.7)	0.43

COVID‐19 patients			
LV function			
LVEF, % (*n* = 58)	56.0 (50.0; 60.0)	57.6 (49.8; 62.3)	0.28
GLS, % (*n* = 70)	17.4 (14.9; 19.6)	16.4 (13.4; 17.7)	0.077
E/e′ ( *n* = 62)	10.2 (8.4; 13.1)	12.3 (7.7; 18.3)	0.65
RV function			
TAPSE, cm (*n* = 68)	2.05 (1.80; 2.30)	1.93 (1.56; 2.39)	0.20
RVLS, % ( *n* = 18)	23.7 (20.8; 29.0)	20.3 (7.2; 29.8)	0.31
TR V_max_ , m/s ( *n* = 24)	2.1 (1.5; 2.8)	2.7 (2.3; 2.9)	0.073
Cardiac biomarkers			
Troponin > ULN, % (*n* = 30)	3 (20%)	6 (40%)	0.23
NT‐proBNP, pg/mL ( *n* = 28)	618 (168; 1903)	665 (154; 2246)	0.61

Abbreviations: E/e′, early diastolic peak mitral inflow velocity to early diastolic peak tissue velocity; GLS, global longitudinal strain; LV, left ventricular; LVEF, left ventricular ejection fraction; NT‐proBNP, N‐terminal pro‐brain natriuretic peptide; RV, right ventricular; RVLS, right ventricular free wall longitudinal strain; TAPSE, tricuspid annular plane systolic excursion; TR V_max_, tricuspid regurgitation peak velocity; ULN, upper limit of normality.

### 3.2 Comparison to Hospitalized COVID‐19 Patients

All 36 FluHeart participants were successfully matched 1:1 to 36 hospitalized COVID‐19 patients. Median time from admission to echocardiography varied significantly between cases and controls (1 day [IQI: 1; 1] vs. 4 days [IQI: 2; 9], *p* < 0.001). Influenza patients were more likely to have a history of smoking and higher systolic blood pressure but less likely to use cholesterol‐lowering medication. However, no difference was observed in early warning scores at the exam or CRP levels. When evaluating cardiac parameters, no differences in LV function, RV function, or cardiac biomarker levels were observed between influenza and COVID‐19 patients. Echocardiographic and cardiac biomarker parameters for each group are detailed in Table 3, and the mean differences in echocardiographic measures are depicted in Figure 1.

## 4 Discussion

This interim analysis of the FluHeart study, investigating hospitalized patients with laboratory‐confirmed influenza through echocardiography and cardiac biomarkers, revealed a substantial prevalence of cardiac dysfunction. Among the participants, a majority exhibited LV dysfunction, whereas RV dysfunction was less frequent. Additionally, biochemical assessments revealed levels of myocardial injury and acute heart failure comparable to the incidence of RV dysfunction. Both LV and RV function was significantly worse than in healthy matched controls from the general population but similarly impaired when compared to matched hospitalized COVID‐19 patients.

This is the first prospective study using echocardiography to assess cardiac function among hospitalized patients with laboratory‐confirmed influenza. Notably, no previous study has leveraged speckle‐tracking echocardiographic analysis, which allows for a more sensitive assessment of cardiac function than conventional echocardiographic methods [19]. In 2021, Al Saud et al. [13] published a prospective cohort study involving 117 adult and pediatric patients presenting to the emergency department with fever and respiratory symptoms. All participants were tested for influenza and examined with ultrasound of the heart; however, cardiac function was only estimated visually. Among the subset of 49 patients testing positive for influenza, mean age was 42 ± 30 years, 28 were females, and 28 were subsequently hospitalized. Within this group, LVEF was estimated to be less than 50% in 4, and RV dysfunction was observed in 1. Al Saud et al. found that LV and RV dysfunction were significantly less prevalent compared to the current study. However, there were fundamental differences between these two studies, including the sample of patients (younger patients presenting to the emergency department vs. older hospitalized patients) and evaluation method (simple visual evaluation vs. quantitative echocardiographic analyses), which limits direct comparisons of these two studies. Mizera et al. [12] conducted the only other study to assess cardiac findings during acute seasonal influenza with echocardiography. The authors compared 150 prospectively included hospitalized adult COVID‐19 patients with 150 age‐ and sex‐matched retrospectively included hospitalized adult influenza patients (mean age 68 ± 15 years, 88 males). The study reported similar echocardiographic findings to the present study. In influenza patients, mean LVEF was 54% with impairment in 25% of patients (although the definition of impairment was not stated). Mean TAPSE was 2.1 cm, and RV dysfunction was visually observed in 18% of patients. Mizera et al. found that the prevalence of impaired LVEF was higher in influenza patients when compared to COVID‐19 patients. However, no difference was observed in continuous LVEF, TAPSE, or visual RV dysfunction, consistent with the findings of the present study. Furthermore, pulmonary arterial systolic pressure was higher in COVID‐19 patients, which we similarly observed in this study when comparing TR V_max_; however, the difference was not statistically significant. Although the findings of Mizera et al. are partially consistent with the results of the present study, it is important to consider the significant selection bias inherent in retrospective studies as the one conducted by Mizera et al. This bias may account for key differences in the findings, such as the higher prevalence of impaired LVEF in retrospective influenza patients compared to prospective COVID‐19 patients.

It is likely that a combination of systemic complications [20, 21, 22, 23, 24, 25, 26, 27, 28, 29, 30] to influenza may collectively contribute to impaired cardiac function: secondary pulmonary hypertension, increased myocardial oxygen demand, hypoxemia, hypotension, and a prothrombotic state, along with suggested direct effects [31, 32, 33, 34, 35, 36] such as myocardial inflammation and plaque instability due to cardiac virus relocation. However, we cannot dismiss the possibility of a higher prevalence of subclinical heart disease in influenza patients requiring hospitalization, a group that may be more susceptible to a worse clinical course as reported previously [37]. The observation of concurrent myocardial injury assessed through hs‐TnI suggested an acute component of myocardial stress in these patients. This is reinforced by the finding that all patients with elevated hs‐TnI exhibited either LV or RV dysfunction. Regardless of the underlying cause, a substantial prevalence of impaired cardiac function (in both ventricles) was observed among hospitalized influenza patients. Due to the impracticality of conducting a study involving echocardiography prior to infection to ascertain whether cardiac impairment preexisted, alternative approaches are necessary to investigate the underlying nature of diminished cardiac function. In this regard, reexamination of all surviving participants of the FluHeart study 2–3 months post‐hospital discharge was planned. These findings, to be reported upon study finalization, may provide valuable insights toward addressing this question.

Evaluating the clinical implications of echocardiography‐assessed cardiac dysfunction in hospitalized influenza patients would require a larger sample size to ensure sufficient events for meaningful analysis. Such objectives will be explored in the final study sample of the FluHeart study. However, impaired echocardiographic parameters of cardiac function have been linked to acute respiratory distress syndrome and mortality among hospitalized COVID‐19 patients [38, 39]. These findings may be applicable to a similarly infected and respiratory‐compromised population, such as those hospitalized for influenza if these impairments are primarily due to systemic complications to infection or a indicate preexisting subclinical cardiac dysfunction in frailer individuals. Nonetheless, it is premature to recommend changes in clinical management based solely on abnormal echocardiographic parameters. Clinicians should continue to provide usual care for influenza patients presenting with symptoms of cardiac disease or are hemodynamically compromised.

### 4.1 Limitations

The results should be considered hypothesis‐generating only, given the observational nature of this study, and interpreted with additional caution due to several limitations in this interim analysis that warrant consideration. First, the sample size was relatively modest, rendering the study susceptible to Type 2 errors due to limited statistical power and to the potential effects of sampling errors, which may, for instance, explain the low number of patients with heart failure. Nonetheless, it is important to highlight that age and sex distribution were comparable between included subjects and screen failures. Unfortunately, we were unable to record comorbidities among screen failures, so we cannot rule out the possibility of residual selection bias. The limited number influenza patients constrained the number of factors upon which controls could be matched upon. Notably, there was a significant difference in the prevalence of chronic obstructive pulmonary disease and/or asthma between influenza patients and controls from the general population. As chronic obstructive pulmonary disease is predominantly associated with worse right ventricular function [40], this difference may partly explain some of the differences observed between these two groups. Furthermore, median time from admission to study exam differed by 3 days between influenza patients and the matched COVID‐19 patients, potentially raising concerns regarding comparability. However, it is important to note that markers of disease severity (such as CRP and early warning score) were similar between these two groups, limiting the potential influence of this difference. Finally, the quality of echocardiographic examinations in influenza and COVID‐19 patients was naturally limited, as they were performed bedside on respiratory‐compromised patients who were often suboptimally positioned, resulting in fewer available RVLS measurements.

## 5 Conclusion

In this interim analysis of the FluHeart study, a substantial prevalence of both LV and RV dysfunction was observed among hospitalized influenza patients. Notably, RV and LV function measures were significantly worse in these patients than matched controls. The level of impairment resembled that observed in hospitalized COVID‐19 patients.

## Author Contributions

K.G.S., D.M., L.N., J.U.S.J., and T.B.S. have contributed significantly to the conceptualization and design of the FluHeart study. Data collection for the FluHeart study was carried out by K.G.S., F.S.D., E.D., M.C.H.L., M.D., A.M.R.J., N.D.J., M.S., F.V., and N.E.L. P.S., R.M., and T.B.S. were responsible for the 5th Copenhagen City Heart Study, where K.G.S., M.C.H.L., N.D.J., and R.H. carried out data collection. K.G.S., M.C.H.L., and T.B.S. was responsible for the ECHOVID‐19 study, where data collection was carried out by K.G.S., F.S.D., M.C.H.L., N.D.J., A.B.N., and J.C. Access to and analysis of the raw data was carried out by K.G.S. and T.B.‐S. The initial draft of the manuscript was prepared by K.G.S., and all authors participated in reviewing the draft and endorsing the final manuscript.

## Ethics Statement

This study was approved by a regional ethics committee.

## Consent

Informed consent was obtained from all.

## Conflicts of Interest

K.G.S. reports: Advisory Board: Sanofi Pasteur. T.B.S. reports: chief investigator of the Boston Scientific financed “DANLOGIC‐HF” trial; chief investigator of the Sanofi Pasteur financed “NUDGE‐FLU” trial; chief investigator of the Sanofi Pasteur financed “DANFLU‐1” trial; chief investigator of the Sanofi Pasteur financed “DANFLU‐2” trial; Steering Committee member of Boston Scientific sponsored “LUX‐Dx TRENDS Evaluates Diagnostics Sensors in Heart Failure Patients Receiving Boston Scientific's Investigational ICM System” trial; Steering Committee member of the Amgen sponsored GALACTIC‐HF trial; Steering Committee member of the Boehringer Ingelheim financed EASi‐KIDNEY trial; Advisory Board: Sanofi Pasteur, Amgen, CSL Seqirus, and GSK; Speaker Honorarium: Bayer, Novartis, Sanofi Pasteur, GE Healthcare, and GSK; research grants: Boston Scientific, GE Healthcare, AstraZeneca, Novo Nordisk, and Sanofi Pasteur; consultant appointments: Novo Nordisk, IQVIA, and Parexel. The other authors declare no conflicts of interest.

## Data Availability

The data underlying this article cannot be shared publicly due to Danish and European data laws. The data will be shared on reasonable request to the corresponding author.
